# Characterization of primary cilia features reveal cell-type specific variability in in vitro models of osteogenic and chondrogenic differentiation

**DOI:** 10.7717/peerj.9799

**Published:** 2020-08-21

**Authors:** Priyanka Upadhyai, Vishal Singh Guleria, Prajna Udupa

**Affiliations:** Department of Medical Genetics, Kasturba Medical College, Manipal, Manipal Academy of Higher Education, Manipal, Karnataka, India

**Keywords:** Primary cilia, Osteogenic differentiation, Chondrogenic differentiation, C3H10T1/2, MC3T3-E1, ATDC5, Cilia length and frequency alteration

## Abstract

Primary cilia are non-motile sensory antennae present on most vertebrate cell surfaces. They serve to transduce and integrate diverse external stimuli into functional cellular responses vital for development, differentiation and homeostasis. Ciliary characteristics, such as length, structure and frequency are often tailored to distinct differentiated cell states. Primary cilia are present on a variety of skeletal cell-types and facilitate the assimilation of sensory cues to direct skeletal development and repair. However, there is limited knowledge of ciliary variation in response to the activation of distinct differentiation cascades in different skeletal cell-types. C3H10T1/2, MC3T3-E1 and ATDC5 cells are mesenchymal stem cells, preosteoblast and prechondrocyte cell-lines, respectively. They are commonly employed in numerous in vitro studies, investigating the molecular mechanisms underlying osteoblast and chondrocyte differentiation, skeletal disease and repair. Here we sought to evaluate the primary cilia length and frequencies during osteogenic differentiation in C3H10T1/2 and MC3T3-E1 and chondrogenic differentiation in ATDC5 cells, over a period of 21 days. Our data inform on the presence of stable cilia to orchestrate signaling and dynamic alterations in their features during extended periods of differentiation. Taken together with existing literature these findings reflect the occurrence of not only lineage but cell-type specific variation in ciliary attributes during differentiation. These results extend our current knowledge, shining light on the variabilities in primary cilia features correlated with distinct differentiated cell phenotypes. It may have broader implications in studies using these cell-lines to explore cilia dependent cellular processes and treatment modalities for skeletal disorders centered on cilia modulation.

## Introduction

Primary cilia are non-motile sensory organelles that protrude from most mammalian cell surfaces (Singla & Reiter, 2006). They transduce various extracellular cues essential for proliferation, differentiation and homeostasis via Hedgehog (Hh), Wnt, Notch, Receptor Tyrosine Kinase, Transforming growth factor β (TGFβ), G protein-coupled receptor and calcium signaling pathways (Christensen et al., 2012; Clement et al., 2013; Ezratty et al., 2011; Huangfu et al., 2003; Lancaster, Schroth & Gleeson, 2011; Nauli et al., 2003; Wheway et al., 2015). The importance of cilia in regulating embryonic and postnatal development is underscored by a wide spectrum of human disorders termed as ciliopathies caused by aberrations in cilia formation and function.

The ciliary backbone or axoneme is a microtubular structure that emanates from a modified centriole, the basal body within the ciliary pocket and is encased in a ciliary membrane contiguous with the plasma membrane of the cell. The transport of proteins into the cilia and the bidirectional intra-ciliary movement of cargo is driven by the intraflagellar transport (IFT) machinery composed of multimeric protein complexes, including but not limited to IFT-A/B complexes and associated motor-proteins, kinesin-2 and dynein 2 (Berbari et al., 2008; Cole et al., 1998; Follit et al., 2006; Nonaka et al., 1998; Omori et al., 2008; Pazour, Wilkerson & Witman, 1998; Piperno & Mead, 1997; Porter et al., 1999; Signor et al., 1999; Snow et al., 2004; Yoshimura et al., 2007). This not only facilitates the receipt and transduction of signals at the primary cilium but is required for its assembly and maintenance (Bhogaraju et al., 2013; Cole et al., 1998; Wren et al., 2013).

A growing body of evidence has revealed that bone and cartilage are dynamic entities that assimilate a broad range of sensory cues to facilitate skeletal formation and repair. This is augmented by primary cilia extending from the cell surface of osteoblasts, osteocytes, chondrocytes and their precursor preosteoblast and mesenchymal stem cells (MSCs) (Federman & Nichols, 1974; Scherft & Daems, 1967; Tummala, Arnsdorf & Jacobs, 2010). Cilia serve as critical mechanosensors of fluid flow in osteoblasts and osteocytes (Temiyasathit et al., 2012), whereas in chondrocytes their main function is sensing peripheral tissue deformation (McGlashan, Jensen & Poole, 2006). In this context mechanical stimulation serves as a crucial anabolic signal; it promotes osteogenic (OS) differentiation in MSCs and mineralization of the chondrocyte matrix (Chen et al., 2015; Hoey, Kelly & Jacobs, 2011; O’Conor et al., 2014). Primary cilia are also required for osteoblast, osteocyte polarity, alignment in bone development and regulate chondrocyte cell polarity at the growth plate (Lim et al., 2020a; Song et al., 2007). They are chemosensitive and serve as a nexus of signaling activity important for OS and chondrogenic (CH) differentiation, such as Hh, TGFβ, Fibroblast growth factor (FGF), Wnt, platelet derived growth factor and parathyroid hormone related peptide (Cai et al., 2012; Caplan & Correa, 2011; Chen, Deng & Li, 2012; Jiang et al., 2016; Kozhemyakina, Lassar & Zelzer, 2015). The essential role of ciliary function in osteoblast and chondrocyte differentiation is substantiated by impaired differentiation following cilia abrogation caused by knockdown of constituents of the IFT machinery, for example, Ift80, Ift88 and Kif3a (Qiu et al., 2012; Tummala, Arnsdorf & Jacobs, 2010; Wang, Yuan & Yang, 2013; Yang & Wang, 2012). Moreover, many ciliopathies manifest with altered cilia length and preponderance, underscoring the importance of cilia in cellular function (Failler et al., 2014; Zhang et al., 2016).

The basal levels of ciliation and cilia length have been described for several skeletal cell-lines, for example, MC3T3-E1, MLO-Y4, chondrocytes and mesenchymal stem cells (Brown et al., 2014; Malone et al., 2007; Wann & Knight, 2012; Xiao et al., 2006). Ciliary attributes may be tailored in accordance with distinct differentiated cell states. This is corroborated by a previous study that demonstrated lineage specific changes in primary cilia length and frequencies in response to chemically induced differentiation in human MSCs (hMSCs) over a 7 day period (Dalbay et al., 2015). Primary cilia length are malleable and modulated by several factors such as cytoskeletal actin organization and inflammatory cytokines (Kim et al., 2010; McMurray et al., 2013; Pitaval et al., 2010; Wann & Knight, 2012). Modulation of the anterograde IFT transport speed by intracellular cyclic AMP levels have also been shown to control ciliary length (Besschetnova et al., 2010). In chondrocytes compressive loading was reported to reversibly reduce cilia length and incidence (McGlashan et al., 2010). Moreover, variable stimulation with growth factors may produce disparate consequences on cilia length. While the constitutive activation of FGF signaling was shown to cause primary cilia shortening, its transient stimulation resulted in cilia elongation, in growth plate chondrocytes and limb bud derived mesenchymal cells (Kunova Bosakova et al., 2018; Martin et al., 2018).

C3H10T1/2, MC3T3-E1 and ATDC5 are murine mesenchymal stem cells, preosteoblast and prechondrocyte cell-lines, respectively, that are commonly employed in various in vitro studies, interrogating the mechanisms underlying osteoblast and chondrocyte differentiation, bone disorders and repair (Hino et al., 2018; Lee et al., 2017; Wang et al., 2019; Yao & Wang, 2013). Despite the wide use of these cell-lines, how ciliary features vary in them during native OS and CH differentiation has not been so far investigated. Here we evaluate the primary cilia length and frequency during OS differentiation in C3H10T1/2 and MC3T3-E1 and CH differentiation in ATDC5 cells, over a period of 21 days. Our study revealed cell-type and lineage specific modulation of ciliary characteristics during extended periods of differentiation. These findings expand our existing knowledge and shine light on the primary cilia features in these cell-lines correlated with distinct differentiated cell fates, and may be significant for clinically relevant and explorative studies evaluating cilia dependent molecular mechanisms in these cellular models.

## Materials and Methods

### Cell culture

ATDC5 cell-line (gift from Dr. Uwe Kornak, Charité—Universitätsmedizin Berlin) were propagated in complete media consisting of DMEM/F-12 (1:1) with 1% L-Glutamine, 10% heat inactivated fetal bovine serum (FBS), one mM sodium pyruvate, 100 U/ml penicillin and 100 μg/ml streptomycin (HiMedia, Mumbai, India). Cells were induced with CH media comprising of complete media supplemented with 10^−7^ M dexamethasone, 50 μg/ml ascorbic acid, 10 mM β glycerophosphate and 1X insulin-transferrin-sodium selenite supplement (all from Sigma–Aldrich, St. Louis, MO, USA). Current method of CH differentiation was adapted from previous studies (Newton et al., 2012; Weiss et al., 2012). C3H10T1/2 was obtained from the cell repository at National Center for Cell Science, India and MC3T3-E1, sub-clone 4 was a gift from Dr. Uwe Kornak, Charité—Universitätsmedizin Berlin and were cultured in complete media comprising of DMEM and modified Eagle’s minimum essential medium, respectively, with 1% L-Glutamine, 10% heat inactivated FBS, one mM sodium pyruvate, 100 U/ml penicillin and 100 μg/ml streptomycin (HiMedia, Mumbai, India). OS media comprised of complete media supplemented with 10^−7^ M dexamethasone, 50 μg/ml ascorbic acid and 10 mM β glycerophosphate. All cells were incubated in a humidified atmosphere (37 °C, 5% CO_2_) and media was replaced every second or third day for 21–30 days, as indicated.

### Alizarin red staining

Calcium deposition in the extracellular matrix (ECM) was estimated by Alizarin red dye that combines with calcium in the cellular matrix, as described previously (Ovchinnikov, 2009; Yamakawa et al., 2003). Briefly, cells were seeded in triplicates at the following densities: 18,000/cm^2^ (C3H10T1/2), 11,000/cm^2^ (MC3T3-E1) and 12,000/cm^2^ (ATDC5), respectively in multi-well plates, and were induced ~24 h later with OS or CH differentiation media. Uninduced cells were propagated in complete media for the same period of time as those induced. At 7, 14 and 21 days post differentiation, cells were fixed in 4% paraformaldehyde (PFA) and stained with 2% Alizarin red, pH 4.2 (Sigma–Aldrich, St. Louis, MO, USA) for 40 min at room temperature (RT). Stained monolayers were washed with distilled water and images were captured with inverted phase contrast microscope (Olympus, Tokyo, Japan). Levels of mineralization were quantified by extraction of the stain using 10% (v/v) acetic acid and absorbance was measured at 405 nm (Gregory et al., 2004).

### Alkaline phosphatase staining

C3H10T1/2 and MC3T3-E1 cells were seeded in triplicates at 18,000/cm^2^ and 11,000/cm^2^ per well, respectively in multi-well plates. They were induced ~24 h later with OS differentiation media or left uninduced for the same duration as those induced. At 7, 14 and 21 days post differentiation, cells were fixed in 4% PFA, and alkaline phosphatase (*Alp*) was detected by incubating with nitro blue tetrazolium (NBT)/5-bromo-4-chloro-3-indolyl phosphate substrate (Sigma–Aldrich, St. Louis, MO, USA) for up to 30 min at RT. After washing the stained cells with 1X PBS, images were captured using inverted phase contrast microscope (Olympus, Tokyo, Japan).

### Alcian blue staining

Glycosaminoglycan (GAG) deposition in the ECM was ascertained following CH differentiation in ATDC5 cells plated at a density of 12,000/cm^2^. Subsequently, the cells were fixed in 95% methanol, followed by incubation in 0.1 M HCl and stained with 1% Alcian blue 8GX solution in 3% acetic acid (Sigma–Aldrich, St. Louis, MO, USA). Stained cells were washed with distilled water and images were captured with inverted phase contrast microscope (Axiovert A1 FL; Zeiss, Oberkochen, Germany). Staining intensity was estimated using Fiji (http://imagej.net/Fiji) and represented in arbitrary units.

### Primary cilia detection during osteogenic and chondrogenic differentiation

For detection of cilia by immunostaining, cells were seeded on glass coverslips in multi-well plates at the following densities: 18,000/cm^2^ (C3H10T1/2), 11,000/cm^2^ (MC3T3-E1) and 12,000/cm^2^ (ATDC5). They were induced ~24 h later with OS (C3H10T1/2 and MC3T3-E1) or CH (ATDC5) differentiation media and propagated for 7, 14 and 21 days. The uninduced cells were propagated in complete media for the same period of time. To enhance ciliogenesis, all cells were serum starved (ss) as described earlier (Prosser & Morrison, 2015). To this end induced and uninduced cells were cultured in differentiation or complete media containing 0.5% FBS for 48 h prior to staining. Two sets of cells were fixed prior to differentiation: one was starved (day 0 uninduced), while the other remained non-starved (day 0 uninduced no ss).

### Immunocytochemistry and image analyses

The cells were fixed in 4% PFA, permeabilized in 0.2% Triton X-100, blocked in 5% normal goat serum and finally incubated overnight at 4 °C with primary antibodies—anti-acetylated α tubulin (mouse monoclonal, 1:4,000, cat# T7451; Sigma–Aldrich, St. Louis, MO, USA) and anti-Arl13B (rabbit polyclonal, 1:2,000, cat# 17711-1-AP; ProteinTech, Rosemont, IL, USA) diluted in blocking solution. Cells were incubated in the following secondary antibodies: Alexa fluor 488 goat anti-rabbit and Alexa fluor 568 goat anti-mouse IgG (cat.# A11034 and cat.# A11031; Molecular Probes, Eugene, OR, USA/ThermoFisher Scientific, Waltham, MA, USA), diluted at 1:500 for 2 h at RT. Nuclei were stained using DAPI and mounted in Prolong Diamond Antifade mountant (both from Invitrogen, Carlsbad, CA, USA; ThermoFisher Scientific, Waltham, MA, USA). Images were acquired using an inverted fluorescence microscope equipped with LD Plan-Neofluar 63X/0.75 Corr Ph2 oil immersion objective and Axiocam 503 CCD camera (Axiovert A1 FL; Zeiss, Oberkochen, Germany). Primary cilia were discerned by acetylated α tubulin or Arl13B staining and their length were determined manually by tracing along them using Fiji (http://imagej.net/Fiji). Cilia lengths are represented in micrometer (μm). Immunolabeled entities with a minimal length of 1.5 μm were ascertained as primary cilia. Overall length was assessed for a total of 100–178 cilia in three independent replicates. Ciliary frequencies were evaluated in ~100–200 cells per condition in each of three independent replicates.

### Quantitative real time PCR analyses

Cells were seeded at the following densities for RNA extraction: 21,000/cm^2^ (C3H10T1/2), 15,000/cm^2^ (MC3T3-E1) and 17,000/cm^2^ (ATDC5). They were induced ~24 h later with OS (C3H10T1/2 and MC3T3-E1) or CH (ATDC5) differentiation media and propagated for 7, 14 and 21 days. The uninduced cells they were propagated in complete media for the same period of time. All cells were starved as described above. Total RNA was extracted using Trizol reagent (Invitrogen, Carlsbad, CA, USA; ThermoFisher Scientific, Waltham, MA, USA). For each sample, total RNA content was assessed by absorbance at 260 nm and purity by A260/280 ratios, and then reverse transcribed using Superscript IV Vilo mastermix™ (Invitrogen, Carlsbad, CA, USA; ThermoFisher Scientific, Waltham, MA, USA) according to the manufacturer’s protocol. Real time quantitative PCR was carried out using StepOne (Applied Biosystems, Foster City, CA, USA; ThermoFisher Scientific, Waltham, MA, USA) with a final reaction volume of 10 μl. All reactions were prepared with five μl of 2x PowerUP™ SYBR™ Green Mastermix (Applied Biosystems, Foster City, CA, USA; ThermoFisher Scientific, Waltham, MA, USA), and run in duplicates for each of three independent replicates. The mRNA levels for target genes were normalized to *GAPDH* using primer sequences indicated in Table 1. Quantification was carried out using the ΔΔCt method.

**Table 1:** Primers used in qRT-PCR (F: forward; R: reverse).

Gene	Primer sequence (5′–3′)	Tm (°C)	Product size (bp)
*Alp*	F: CCAACTCTTTTGTGCCAGAGA	58	110
	R: GGCTACATTGGTGTTGAGCTTTT	60	
*Runx2*	F: CTTTACCTACACCCCGCCAG	60	116
	R: GTCCACTCTGGCTTTGGGAA	60	
*Ptch1*	F: CCAGCGGCTACCTACTGATG	60	150
	R: TGCCAATCAAGGAGCAGAGG	60	
*Sox9*	F: TGAAGAACGGACAAGCGGAG	60	198
	R: CAGCTTGCACGTCGGTTTTG	60	
*Mmp13*	F: GGAGCCCTGATGTTTCCCAT	60	165
	R: ATCAAGGGATAGGGCTGGGT	60	
*Gapdh*	F: CATGGCCTTCCGTGTTCCTA	60	172
	R: GTTGAAGTCGCAGGAGACAAC	60	

### SAG mediated modulation of Hedgehog signaling

For stimulation with Smoothened (Smo) Agonist (SAG), cells were seeded as described above and propagated in either complete or OS media supplemented with SAG at a final concentration of one μm. Culture media was replaced every second or third day during the period of induction. Cells were ss for 48 h as mentioned above prior to harvesting.

### Statistical analyses

Data analyses was performed using GraphPad Prism (v8.4) (www.graphpad.com/scientific-software/prism/). One-way analysis of variance (ANOVA) was performed for identifying variation in cilia length among induced and uninduced groups. Two-way ANOVA was performed to assess effects of day to day variation on induction. Nested *t*-test was performed to evaluate whether induction has an overall effect on cilia length, considering day-wise variation is nested within the induction effect. Primary cilia length, frequencies and gene expression studies were performed in three independent replicates and *p* < 0.05 was considered statistically significant. Cilia length and frequency values are shown as mean ± SEM.

## Results

### C3H10T1/2, MC3T3-E1 and ATDC5 undergo the anticipated osteogenic and chondrogenic differentiation in vitro

**Figure 1:**
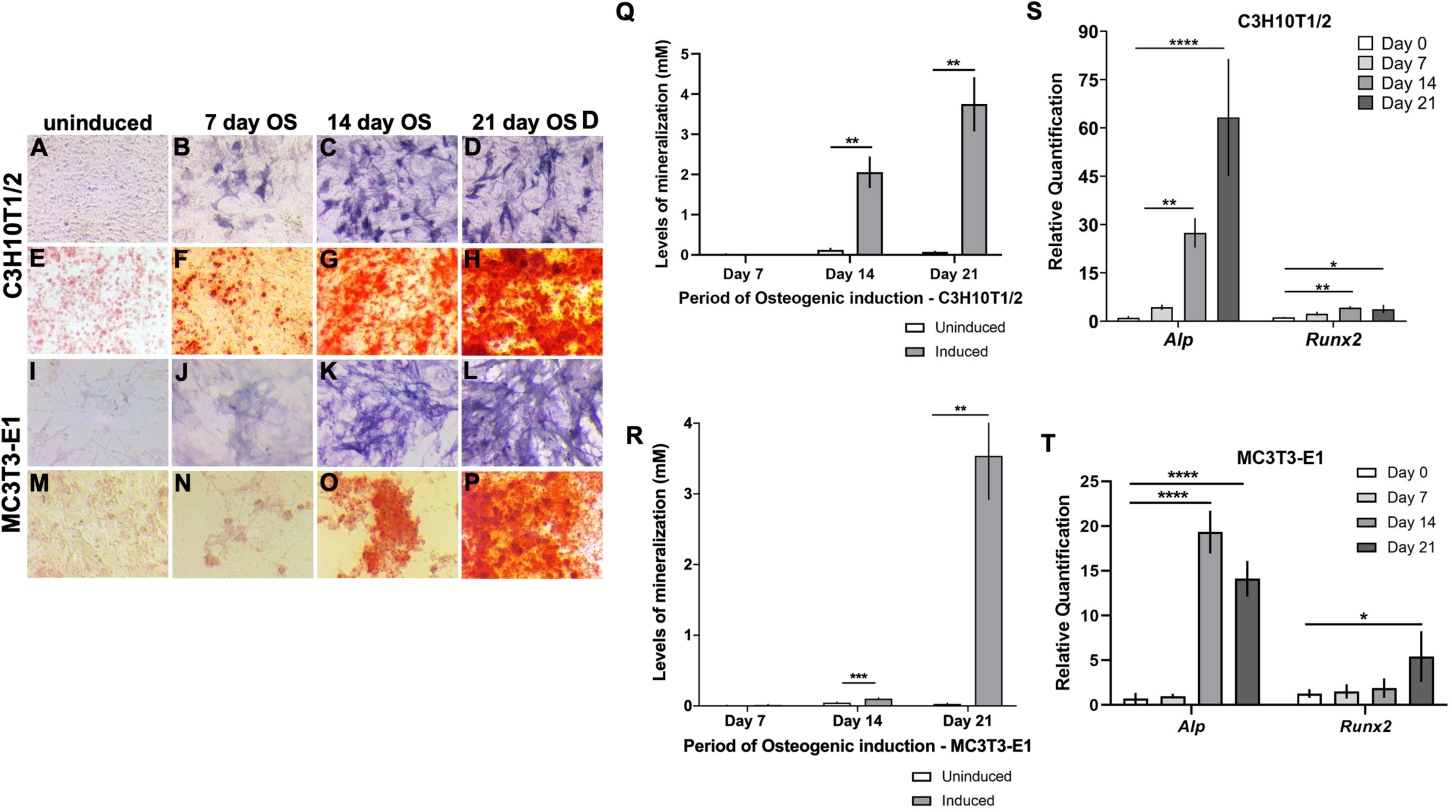
Characterization of in vitro osteogenic differentiation in C3H10T1/2 and MC3T3-E1 cells. (A–P) Enhanced alkaline phosphatase (*Alp*) levels were qualitatively evident in (A–D) C3H10T1/2 and (I–L) MC3T3-E1 cells at 14 and 21 days post osteogenic (OS) induction compared to uninduced. Similarly robust calcium deposition in the ECM was revealed by Alizarin red staining (ARS) in (E–H) C3H10T1/2 and (M–P) MC3T3-E1 cells at 14 and 21 days following OS induction. (Q and R) Quantitative mineralization levels based on ARS confirmed significantly higher extracellular calcium deposition at 14 and 21 days following OS induction compared to day matched uninduced cells in (Q) C3H10T1/2 and (R) MC3T3-E1 cells (**p < 0.01, ***p < 0.001; Two-way ANOVA followed by Sidak’s post hoc test). (S and T) qRT-PCR analyses of the transcript levels of OS differentiation markers, *Alp* and *Runx2*. Levels were normalized to *Gapdh* (*p < 0.05, **p < 0.01, ****p < 0.0001; One-way ANOVA followed by Tukey’s post hoc analyses). (S) In C3H10T1/2, *Alp* and *Runx2* are significantly upregulated at 14 and 21 day OS differentiated cells (T) In MC3T3-E1, *Alp* levels were elevated after 14 and 21 days of OS induction, however, significantly appreciable *Runx2* was detected 21 days following induction.

The deposition of minerals in the form of hydroxyapatite in the ECM is a physiological characteristic of hard tissues such as bone and growth-plate cartilage. *Alp* expressed by osteoblasts and hypertrophic chondrocytes hydrolyzes pyrophosphate to generate inorganic phosphate to promote matrix mineralization (Orimo, 2010). Consequently, high levels of Alp and matrix mineralization reflect osteogenic differentiation. C3H10T1/2 and MC3T3-E1 cells showed elevated OS differentiation following induction from day 14 onward, evidenced qualitatively by pronounced Alp staining in C3H10T1/2 (Figs. 1A–1D) and MC3T3-E1 (Figs. 1I–1L). Elevated calcium deposits in OS induced vs uninduced cells were revealed using Alizarin red staining (ARS) in C3H10T1/2 (Figs. 1E–1H) and MC3T3-E1 (Figs. 1M–1P). Quantitative estimation revealed significant increase in matrix mineralization by day 14 post OS induction that was further enhanced in 21 day induced monolayers (Figs. 1Q and 1R). We evaluated the transcript levels of osteoblast differentiation markers, *Alp* and *Runx2* by qRT-PCR and found that both were significantly upregulated at 14 and 21 days post OS induction in C3H10T1/2 (Fig. 1S). In MC3T3-E1, significantly high *Alp* levels were detected at 14 and 21 days post induction, while elevated *Runx2* was detected at day 21 after OS stimulation (Fig. 1T).

**Figure 2:**
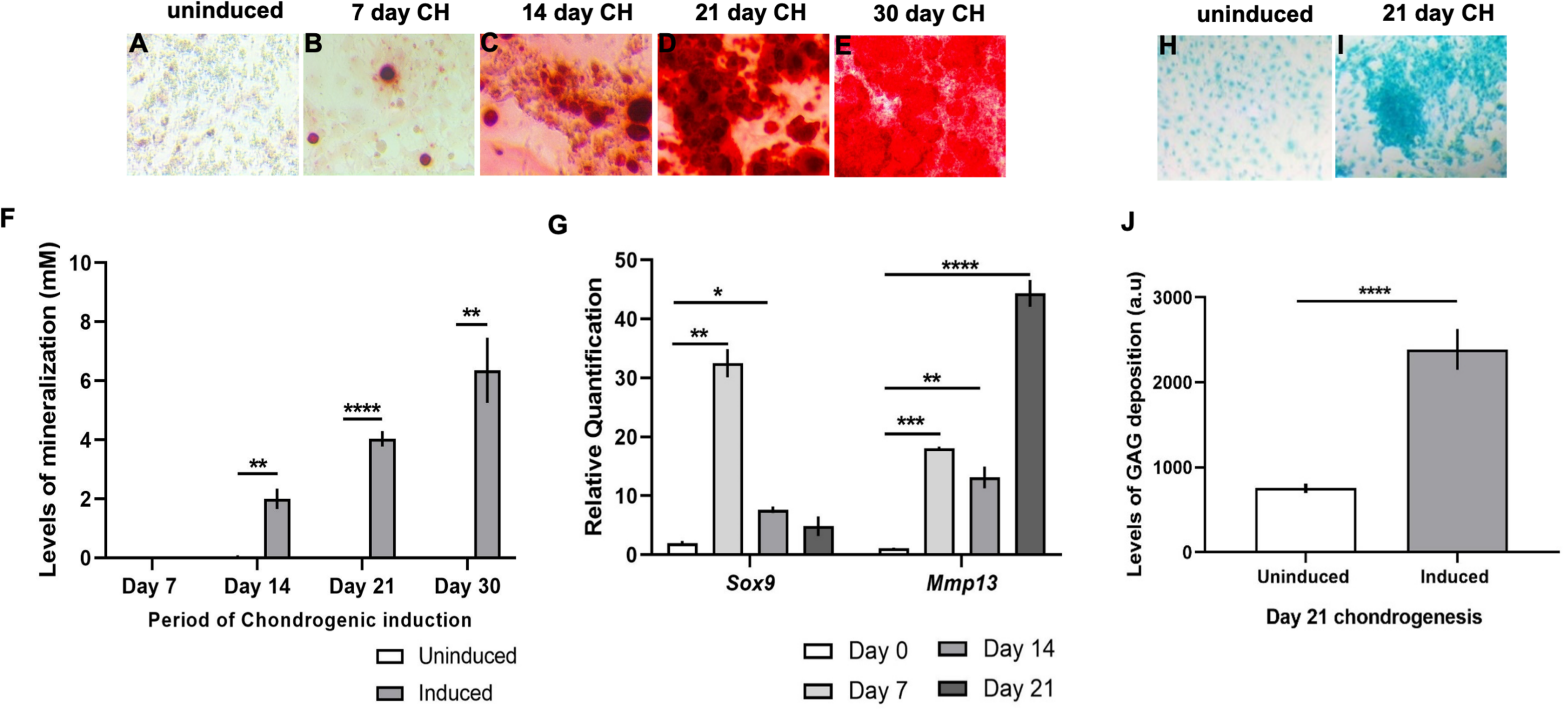
Characterization of chondrogenic differentiation in ATDC5 cells. (A–F) ARS mediated detection of calcium deposition in the ECM following chondrogenic (CH) induction of ATDC5 cells revealed (A–E) perceptible mineralization day 7 onward. (F) Quantitative assessment confirmed significantly high mineralization levels in CH induced monolayers at 14, 21 and 30 days as compared to uninduced cells (**p < 0.01, ****p < 0.0001; Two-way ANOVA followed by Sidak’s post hoc test). (G) qRT-PCR analyses revealed significant upregulation of the transcript levels of CH marker *Sox9* at 7 and 14 days following differentiation (*p < 0.05, **p < 0.01; One-way ANOVA followed Dunnett’s post hoc test), while CH hypertrophy marker *Mmp13* was elevated at 7, 14 and 21 days following differentiation (**p < 0.01, ***p < 0.001, ****p < 0.0001; One-way ANOVA followed by Tukey’s post hoc analyses). Transcript levels were normalized to *Gapdh*. (H–J) Significantly high extracellular glycosaminoglycan (GAG) deposition was detected at 21 days post CH induction (****p < 0.0001; Wilcoxon signed rank test).

ATDC5 cells, when induced for CH differentiation, showed progressive increase in mineral deposition in the ECM from day 14 onward (Figs. 2A–2E), evidenced by quantification of ARS in the induced cellular monolayers as compared to uninduced (Fig. 2F). Transcript levels of *Sox9*, an early marker of CH differentiation was significantly elevated at 7 and 14 days post CH induction and was subsequently diminished at day 21 (Fig. 2G). *Mmp13*, a marker of chondrocyte hypertrophy was upregulated after 7 days of CH media stimulation but was most strongly induced in 21 day CH induced monolayers (Fig. 2G). CH differentiated cells also showed significant GAG deposition in the ECM at 21 days after treatment, revealed by Alcian blue staining (Figs. 2H–2J). Given that cells were seeded at a moderately higher density for gene expression analyses as compared to other assays, we compared cell proliferation using Trypan blue in C3H10T1/2. Cells were seeded at a density of 21,000/cm^2^ on plastic vs 18,000/cm^2^ on glass coverslips, followed by OS induction for 2, 4, 6, 8, 10, 12 days. No significant variability was observed in viable cell numbers between the two substrates (Fig. S1).

### Cilia features demonstrate lineage and cell-type specific alteration in osteogenic and chondrogenic differentiation

We investigated the changes in primary cilia characteristics, for example, length and frequency over 21 days following CH and OS differentiation in ATDC5, C3H10T1/2 and MC3T3-E1 cells. Primary cilia length was discerned following immunolabeling with acetylated α tubulin. To ascertain the reliability of measurement we co-labeled cilia with its markers Arl13b and acetylated α tubulin in a subset of conditions and compared ciliary length detected with each marker in uninduced and induced monoloayers. We found no significant differences in cilia length reported by either marker in these cell-lines (Figs. S2–S4).

#### Chondrogenic induction results in primary cilia elongation in ATDC5 cells

**Figure 3:**
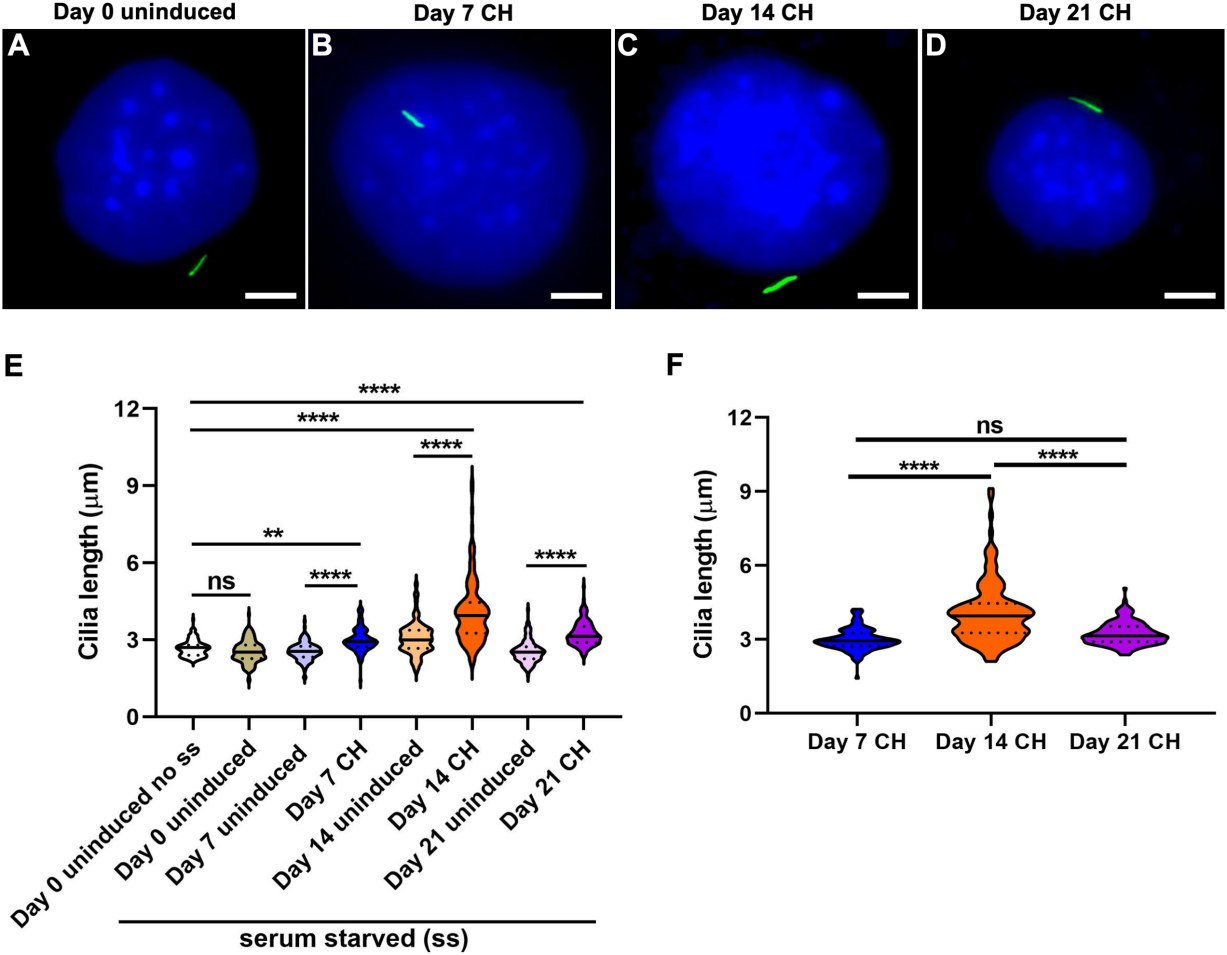
Chondrogenic differentiation causes elongation of primary cilia in ATDC5 cells. Two sets of undifferentiated cells were considered at day 0, namely non-serum starved (day 0 uninduced no ss) and starved (day 0 uninduced). All other CH differentiated and day matched uninduced cells were starved. (A–D) Representative images of ATDC5 primary cilium at day 0 uninduced and days 7, 14 and 21 after CH induction. Primary cilia were labeled with acetylated α tubulin (green), while nuclei were stained with DAPI (blue). Scale bar: five μm. (E) At days 7, 14 and 21 after CH differentiation primary cilia were significantly longer than day 0 and their day matched uninduced control cells. *n* = 110–152 (**p < 0.01, ****p < 0.0001, One-way ANOVA followed by Tukey’s post hoc test). (F) Among the CH induced cells, cilia were longest at day 14 (****p < 0.0001, Two-way ANOVA followed by Tukey’s post hoc test).

Representative images for cells at day 0 uninduced and 7, 14 and 21 days CH differentiated cells are shown (Figs. 3A–3D; Fig. S5). Cilia length was increased at all time points following CH induction compared to day 0 and day matched uninduced controls but were longest after 14 days of differentiation (*n* = 110–152; One-way ANOVA followed by Tukey’s post hoc test; Fig. 3E). Assessing specifically the induced group, showed that cilia in 14 day CH stimulated cells were significantly longer than in 7 and 21 day CH induced monolayers (Two-way ANOVA followed by Tukey’s post hoc test; Fig. 3F). Significant variability in cilia length was noted between day 0 uninduced no ss and day 14 uninduced monolayers (One-way ANOVA followed by Tukey’s post hoc test; Fig. S7A). We also found the CH induction effect to be significantly greater despite day specific variabilities in cilia length (*p* < 0.0001, Nested *t* test). Finally primary cilia frequency did not vary significantly during CH differentiation in ATDC5 cells (Fig. S8A).

#### Osteogenic differentiation was associated with cell-type specific changes in primary cilia length and incidence

**Figure 4:**
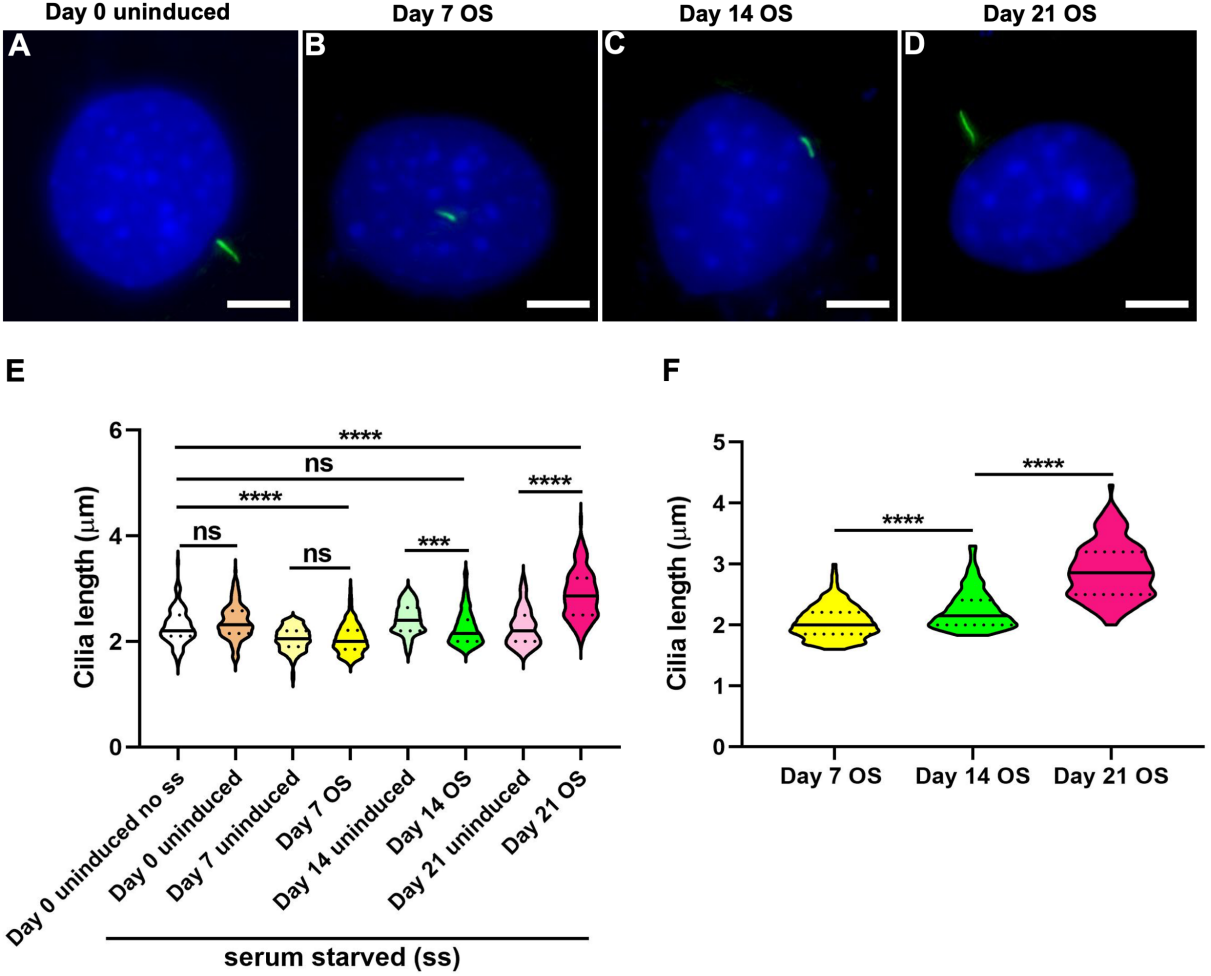
Osteogenic differentiation in C3H10T1/2 mesenchymal stem cells was associated with progressive cilia lengthening. (A–D) Representative images of primary cilium at day 0 uninduced and 7, 14 and 21 days OS induced cells. Primary cilia were labeled with acetylated α tubulin (green), while nuclei were stained with DAPI (blue). Scale bar: five μm. All differentiated and undifferentiated cells were serum starved (ss) except single set of uninduced cells at day 0 (day 0 uninduced no ss). (E) In day 7 OS differentiated cells primary cilia were significantly shorter than in day 0 uninduced; cilia were also shorter after 14 days of OS differentiation but were subsequently elongated in 21 day OS induced cells compared to day 0 and day matched uninduced cells. *n* = 111–177 (***p < 0.001, ****p < 0.0001, One-way ANOVA followed by Tukey’s post hoc test). (F) Among the OS media treated cells, cilia were progressively elongated following differentiation that is, day 7 OS < day 14 OS < day 21 OS (****p < 0.0001, Two-way ANOVA followed by Tukey’s post hoc test).

**Figure 5:**
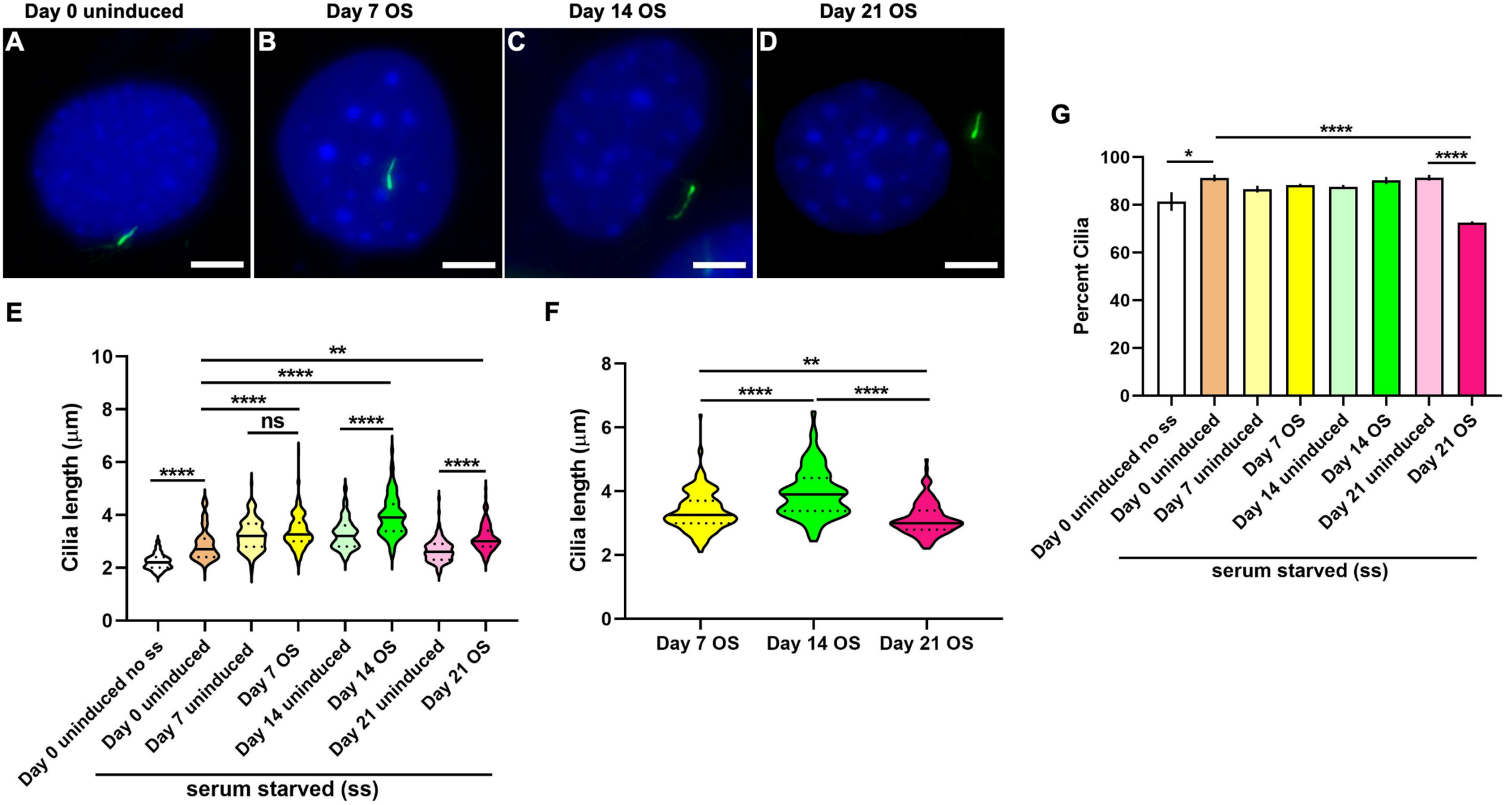
Osteogenic differentiation in MC3T3-E1 preosteoblast cells caused a distinct pattern of cilia elongation and reduction in frequencies. (A–D) Representative images of MC3T3-E1 primary cilium at day 0 uninduced and days 7, 14 and 21 after CH induction. Primary cilia were labeled with acetylated α tubulin (green), while nuclei were stained with DAPI (blue). Scale bar: five μm. All OS treated and untreated cells were serum starved (ss) except one set of uninduced cells at day 0 (day 0 uninduced no ss). (E) Serum starvation caused cilia length increase at day 0; at day 7 after osteogenesis cilia were longer than day 0 uninduced; at 14 and 21 days following OS differentiation the cilia are significantly longer than both day 0 and their corresponding day matched controls. *n* = 101–178 (**p < 0.01, ****p < 0.0001, One-way ANOVA followed by Tukey’s post hoc test). (F) Among OS media induced cells cilia were longest at 14 days of OS differentiation (**p < 0.01, ****p < 0.0001, Two-way ANOVA followed by Tukey’s post hoc test). (G) Starvation appeared to increase primary cilia prevalence in all induced and uninduced monolayers. However, cilia frequencies were significantly reduced only in 21 days OS differentiated cells, *n* = 326–710 (*p < 0.05, ****p < 0.0001; One way ANOVA followed by Tukey’s post hoc test).

C3H10T1/2 and MC3T3-E1 cells were treated to identical OS differentiation conditions. Cell-line specific changes in primary cilia length were noted and representative images are shown (Figs. 4, 5A–5D; Fig. S6). Both cell-lines showed variability in cilia length even in the absence of induction factors (Figs. S7B and S7C).

In C3H10T1/2, primary cilia in 14 day OS differentiated cells were significantly shorter compared to both day 0 and the corresponding day matched uninduced controls, but were longer than in 7 day OS induced cells (*n* = 111–177; One-way ANOVA followed by Tukey’s post hoc test; Fig. 4E). Cilia appeared longest at 21 days post OS differentiation. Overall, cilia seemed to be progressively lengthened with sustained OS differentiation in this cell-line (Two-way ANOVA followed by Tukey’s post hoc test; Fig. 4F). Preponderance of ciliated cells did not vary significantly during OS differentiation in C3H10T1/2 (Fig. S8B).

In MC3T3-E1, serum starvation caused significant increase in primary cilia length and incidence, including in uninduced cells at day 0 (Figs. 5E–5G). Nevertheless, cilia in 14 and 21 days OS media treated cells were significantly elongated, compared to day 0 and day matched uninduced cells (*n* = 101–178; One-way ANOVA followed by Tukey’s post hoc test; Fig. 5E). Among OS media induced MC3T3-E1 cells, cilia were longer after 14 days of OS stimulation compared to 7 and 21 day differentiated cells (Two-way ANOVA followed by Tukey’s post hoc test; Fig. 5F). At 21 days after OS induction, cilia prevalence in MC3T3-E1 was significantly reduced compared to day 0 and day matched uninduced cells, however no differences were observed at other time-points (*n* = 326–710; One-way ANOVA followed by Tukey’s post hoc test; Fig. 5G). For both C3H10T1/2 and MC3T3-E1 despite day-wise cilia length variabilities (Figs. S7B and  S7C) OS induction had a significant effect (*p* < 0.001 and *p* < 0.0001 respectively, Nested *t* test).

### SAG mediated Hedgehog activation is likely associated with increased cilia length and osteoblast differentiation in C3H10T1/2 cells

**Figure 6:**
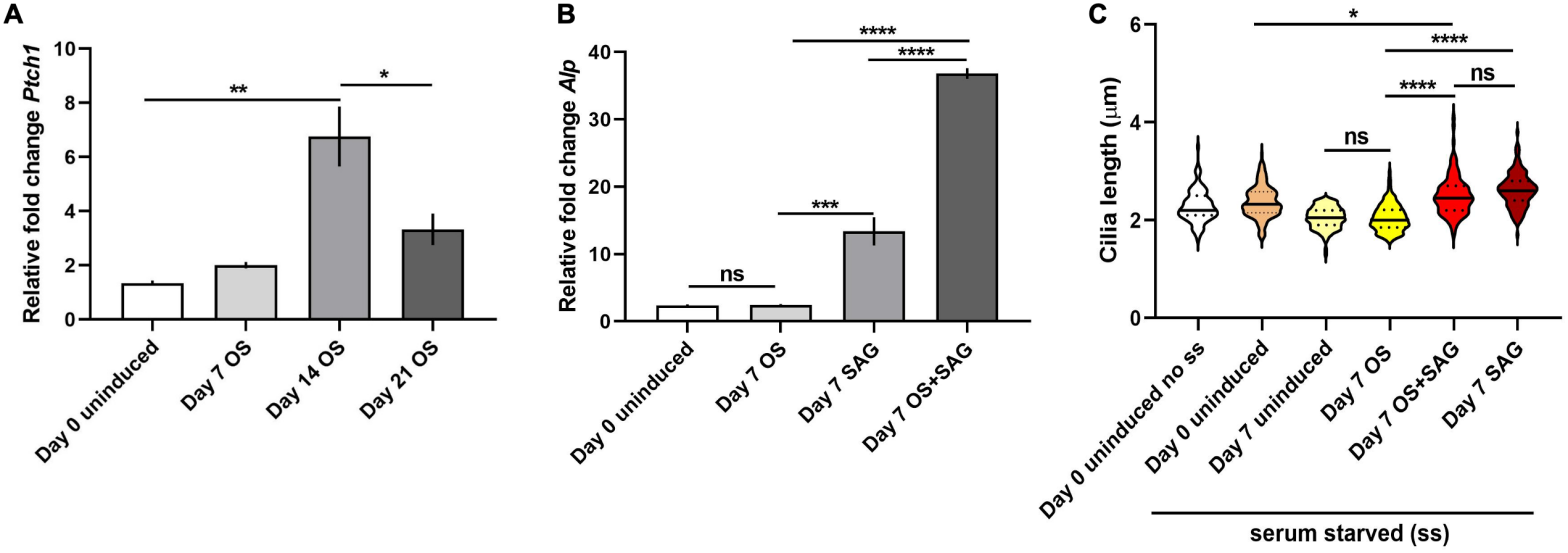
SAG treatment was associated with primary cilia elongation and increased osteoblast differentiation in C3H10T1/2 cells. (A and B) qRT-PCR analyses of the transcript levels of *Ptch1* and *Alp*. Levels were normalized to *Gapdh*. (A) mRNA levels of *Ptch1* was significantly elevated after 14 days of OS induction and then diminished at 21 days (*p < 0.05, **p < 0.01; One-way ANOVA followed by Tukey’s post hoc analyses). (B) Both SAG treatment individually and combined with OS media for 7 days led to significant upregulation of *Alp* transcripts compared to day 7 OS differentiated cells; notably *Alp* levels were more strongly elevated with combinatorial SAG and OS induction as compared to SAG stimulation alone. (***p < 0.001, ****p < 0.0001, One-way ANOVA followed by Tukey’s post hoc test). (C) SAG treatment also resulted in significant elongation of primary cilia compared to day 0 uninduced and 7 day OS differentiated cells. *n* = 105–177 (*p < 0.05, ****p < 0.0001, One-way ANOVA followed by Tukey’s post hoc test).

We assessed the mRNA expression levels of *Patched-1* (*Ptch1*), a direct target of Hh signaling, a canonical ciliary pathway in C3H10T1/2 cells. It was found to be significantly upregulated at 14 days and subsequently diminished at 21 days following OS induction (Fig. 6A). We then tested whether Hh stimulation via treatment with Smo agonist/SAG influenced ciliary characteristics during OS differentiation. SAG treatment alone for 7 days significantly upregulated *Alp* mRNA levels in C3H10T1/2 cells and this effect was further enhanced when combined with OS induction for the same period of time (Fig. 6B). In addition, SAG treatment with or without OS induction produced significant elongation of cilia compared to 7 day OS induced cells (*n* = 105–177; One-way ANOVA followed by Tukey’s post hoc test; Fig. 6C). No change was observed in ciliary frequencies following SAG treatment (Fig. S8C).

## Discussion

Primary cilia are dynamically regulated sensory organelles that integrate diverse stimuli for mediating skeletal development and homeostasis. Ciliary properties, such as length and frequencies are often tightly correlated to cellular function. However, how cilia vary in native OS and CH differentiated monolayers in vitro is less understood. Here we characterized primary cilia features during 21 days of OS and CH differentiation in C3H10T1/2, MC3T3-E1 and ATDC5 cell-lines. In contrast to CH differentiation in hMSCs, which did not alter primary cilia length or shortened them in the presence of TGFβ3 (Dalbay et al., 2015), CH differentiation in ATDC5 cells without TGFβ3 caused cilia elongation (Fig. 3). The mean cilia length observed in non-starved day 0 uninduced ATDC5 cells (2.7 ± 0.03 µm) was similar to that in primary cultures of mouse fetal proliferative chondrocytes (2.82 ± 0.05 µm) but was longer than cilia in proliferative zone of mouse growth-plate cartilage (1.2 ± 0.01 µm) (Martin et al., 2018). CH differentiation was associated with lengthening of primary cilia at all time-points evaluated. Cilia were longest in 14 day CH differentiated cells (4.1 ± 0.11 µm) correlating with intermediate levels of differentiation, compared to those at day 7 (3 ± 0.04 µm) and day 21 (3.2 ± 0.04 µm) post CH induction (Figs. 2 and 3). Highest levels of matrix mineralization, GAG deposition and expression of *Mmp13*, a chondrocyte hypertrophy marker were noted at day 21 post CH induction. The proportion of ciliated non-starved day 0 control ATDC5 cells (83.9 ± 3%) were less compared to mouse fetal chondrocytes (92.6 ± 3.7%) (Martin et al., 2018). During CH differentiation in ATDC5 cells ciliary frequencies were not appreciably different and ranged from ~80% to 88% (Fig. S8A).

Dalbay et al. (2015) further reported that OS differentiation in hMSCs for seven days caused cilia length increase and reduction in frequencies. Primary cilia elongation has also been associated with elevated OS differentiation in other contexts (Zhang et al., 2017). Congruently, we observed cilia lengthening, although in a cell-type specific manner, during OS differentiation. The mean cilia length in non-starved day 0 uninduced MC3T3-E1 preosteoblasts (2.2 ± 0.03 µm) and C3H10T1/2 mesenchymal stem cells (2.3 ± 0.03 µm) were shorter compared to mouse primary osteoblasts (~2.9 µm) (Lim et al., 2020b). Cilia length in MLO-Y4 osteocytes have been reported to range from 2 µm to 4 µm (Xiao et al., 2006). In C3H10T1/2 cilia were longest in 21 day OS induced cells (2.9 ± 0.04 µm); at 14 day OS differentiation cilia (2.3 ± 0.03 µm) remained shorter than in controls but were elongated compared to in 7 days of osteogenesis (2.1 ± 0.02 µm) (Fig. 4). In MC3T3-E1, cilia lengths were significantly high in day 14 (3.9 ± 0.06 µm) and 21 (3.1 ± 0.05 µm) OS differentiated cells vs controls but were longest in the former (Fig. 5). Frequencies of ciliated non-starved day 0 uninduced C3H10T1/2 (85.3 ± 2.6%) and MC3T3-E1 (81.4 ± 3.9%) cells were greater than in mouse primary osteoblasts (~70%) (Lim et al., 2020b). Notably the preponderance of ciliated cells significantly decreased in 21 day OS differentiated MC3T3-E1 cells compared to controls (72.5 ± 0.6%). In contrast, cilia incidence remained unchanged during osteogenesis in C3H10T1/2 and ranged from ~79% to 86% (Fig. S8B). It is noteworthy that significant OS differentiation was first evident after 14 days of OS induction and was highest after 21 days in both cell-lines (Fig. 1). These data reflect the occurrence of distinct cell-type specific molecular machineries that likely function by cilia modulation in disparate ways to elicit similar overall functional responses in each cell-line.

Our current approach of primary cilia length evaluation could be influenced by cells being seeded at a moderately lower density and on glass substrate for cilia immunodetection as compared to plastic for gene expression analyses, as well as cell morphology alterations coincident with differentiation. Furthermore cilia length in cultured cells could be influenced by several factors including differentiation media constituents. For example, dexamethasone that is widely utilized for promoting osteogenesis (Derfoul et al., 2006; Langenbach & Handschel, 2013; Weiss et al., 2012) is a potential enhancer of ciliogenesis and cilia length (Forcioli-Conti et al., 2015; Khan et al., 2016). However, its effect may be dosage dependent. In previous studies, cilia elongation has been observed with one μm and 10 μm dexamethasone with some effect being noted at concentrations as low as 10 nM (Forcioli-Conti et al., 2015; Khan et al., 2016). Despite this, using dexamethasone at identical concentrations to those employed here (100 nM), CH induction produced unaltered or shorter cilia, in hMSCs (Dalbay et al., 2015). This suggests that the inclusion of dexamethasone alone may not account for increased cilia length in all contexts. Testing each induction media component individually will be necessary to discern their role in cilia modulation, if any.

While specific mechanism(s) driving cilia length modulation in response to OS and CH differentiation here are unclear, length increase may be essential to enhance ciliary mechanosensitivity, signal transduction and promoting differentiated cell fates. Cilia elongation involves coordinated ciliary membrane extension and modulation of its composition. Longer cilia could experience greater membrane strain, stimulating opening of stretch-activated ion channels in the ciliary membrane or lead to increased bending energy at their base, triggering mechanotransduction signaling (Resnick, 2015; Resnick & Hopfer, 2007; Schwartz et al., 1997; Spasic & Jacobs, 2017). Increase in cilia length also enhances its surface area leading to accelerated synthesis and trafficking of cilia-specific proteins and signaling molecules to concentrate them in the ciliary microdomain for greater signaling activity (Breslow et al., 2013; Kee et al., 2012).

The Hh pathway performs essential roles in early limb bud patterning, stimulation of osteoblast differentiation in endochondral and intramembranous ossification and postnatal bone homeostasis (Abzhanov et al., 2007; Horikiri et al., 2013; Kronenberg, 2003; Zhu et al., 2008). The expression of Sonic hedgehog (Shh) was shown to be upregulated during OS differentiation in rat MSCs (Ma et al., 2013). In mammals, the cilium forms an indispensable scaffold to concentrate Hh pathway components, modulating responsiveness to its ligands and regulating activator and repressor forms of the Glioma (Gli) family of transcription factors that control the expression of Hh-target genes (Huangfu et al., 2003; Liu, Wang & Niswander, 2005; May et al., 2005). Accordingly, Gli2 and Gli3 that are essential for mouse skeletal development (Hui & Joyner, 1993; Mo et al., 1997) and their proteolytic processing machinery localize at the cilium (Haycraft et al., 2005; Mick et al., 2015). Disruption of ciliary components, Ift80, Ift88 and Kif3a caused Gli2 depletion, in addition to defective OS differentiation, but osteogenesis was rescued by Gli2 overexpression (Haycraft et al., 2007; Koyama et al., 2007; Yang & Wang, 2012). In the present study we determined that the expression of *Ptch1*, a direct target and negative regulator of Hh signaling (Carballo et al., 2018), were increased in 14 day OS differentiated C3H10T1/2 cells but subsequently diminished after 21 days of OS stimulation (Fig. 6A). How increased *Ptch1* produces enhanced OS differentiation at 14 days is unclear but its subsequent decline in 21 day OS induced cells was indicative of potentially high Hh activity and was congruent with the highest levels of osteogenesis observed at this time-point. We treated C3H10T1/2 cells with SAG that can activate Smo, the essential transducer of Shh signaling by promoting its ciliary enrichment (Chen et al., 2002; Frank-Kamenetsky et al., 2002; Rohatgi et al., 2009). Alternatively, SAG may mediate Smo activation in a cilia independent manner (Fan et al., 2014). SAG stimulation individually and in combination with OS media treatment was associated with elevated *Alp* expression and significantly elongated cilia in 7 day OS differentiated cells (Figs. 6B and 6C). This is contrary to studies where SAG treatment in neuronal cell-types did not alter cilia length or neuronal activity (Bansal et al., 2019). Modifying cilia length in neural cell-types also did not alter Hh-dependent patterning (Bangs et al., 2015; Bonnafe et al., 2004). Notably Shh treatment has also been shown to potentiate OS differentiation in MC3T3-E1, C3H10T1/2, and ST2 cells (Spinella-Jaegle et al., 2001; Tian et al., 2012). These observations provide initial evidences likely suggesting that Hh activation could influence primary cilia properties and promote OS differentiation in C3H10T1/2, and mandate further dissection of its mechanistic basis.

In the current study we provide a detailed characterization of cilia features in OS and CH differentiated C3H10T1/2, MC3T3-E1 and ATDC5 cells. All cell-lines evaluated here have been extensively utilized to study molecular processes underlying osteoblast, chondrocyte differentiation and bone disorders, for example, osteoporosis and ciliopathies. It is noteworthy that skeletal ciliopathies such as short-rib thoracic dysplasias and cranioectodermal dysplasias are characterized by changes in primary cilia length and frequencies (Dupont et al., 2019; Walczak-Sztulpa et al., 2010). Moreover, degenerative conditions such as bone aging and osteoporosis are marked by the suppression of autophagy in osteocytes owing to their susceptibility to hypoxia and oxidative stress (Onal et al., 2013). Interestingly, primary cilia length modulation has been suggested to control autophagic activity in some contexts (Pampliega et al., 2013). Thus, modifying cilia length and thereby their sensory properties could serve as an attractive treatment modality in bone disorders. C3H10T1/2, MC3T3-E1 and ATDC5 have also been used in tissue engineering studies evaluating bone healing using various types of biomaterial scaffolds, ultrasound frequencies, modulation of three dimensional cellular environment, static magnetic fields and growth factors (Arosarena et al., 2011; Baudequin et al., 2017; Cicuendez et al., 2017; Martinez Sanchez et al., 2017; Matsumoto et al., 2018; Weiss et al., 2012; Yang et al., 2018). In this premise, our data inform on the presence of stable cilia to orchestrate signaling during extended periods of OS and CH differentiation, in these cells. We report unique cell-line specific ciliary characteristics that could be useful to define lineage specific in vitro differentiation phenotypes. Our findings illustrate the dynamic variability in ciliary attributes in native differentiated cell-states. This can serve as a useful reference for studies using these cell-lines to dissect cilia dependent cellular processes or therapies for skeletal disorders involving cilia regulation. Finally our results shed light on the extent of basal variation in ciliary features in the absence of differentiation that may inform on the utility of these cell-lines and appropriate controls depending upon experimental goals. Overall these findings warrant in-depth delineation of the underlying regulatory mechanisms and signaling events in each cell-type.

## Conclusion

We characterized the variation in primary cilia features, length and frequencies in ATDC5, C3H10T1/2 and MC3T3-E1 cells following CH and OS differentiation over 21 days. Briefly both were associated with elongation of cilia but displayed distinct alterations correlating with unique in vitro differentiated cell states. Reduced cilia frequencies were noted in 21 day OS differentiated MC3T3-E1 cells. Further investigations are needed to uncover the specific molecular processes governing the observed cell-line specific variations in cilia features during differentiation.

## Supplemental Information

10.7717/peerj.9799/supp-1 Supplemental Information 1 Comparative evaluation of cell proliferation in C3H10T1/2. Cells were seeded at a density of 18000 cm^2^ and 21000 cm^2^ on glass and plastic, respectively followed by OS induction. Count of viable cells were estimated by Trypan blue at 2, 4, 6, 8, 10 and 12 days and no significant differences were observed (Two way ANOVA followed by Tukey’s post hoc analysis).

10.7717/peerj.9799/supp-2 Supplemental Information 2 Primary cilia length evaluation in uninduced and CH differentiated ATDC5 cells by dual markers. (A) ****Representative images of primary cilium in ATDC5 at day 0 uninduced without serum starvation (day 0 uninduced no ss), day 0 and 7 uninduced and in 7 day CH differentiated cells. Cilia were co-immunolabeled with dual markers, acetylated α tubulin (red) and Arl13b (green); nuclei were labeled by DAPI (blue). (B) Ciliary length was measured for each marker and condition and no significant differences were observed, n=40 (Welch’s t Test).

10.7717/peerj.9799/supp-3 Supplemental Information 3 Primary cilia length estimation by dual markers in uninduced and OS induced C3H10T1/2 cells. (A) ****Representative images of primary cilium in C3H10T1/2 at day 0 uninduced without serum starvation (day 0 uninduced no ss), day 0 and 7 uninduced and in 7 day OS induced cells. Cilia were co-immunolabeled with markers, acetylated α tubulin (red) and Arl13b (green); nuclei were labeled by DAPI (blue). (B) Ciliary length was measured for each marker and condition and no significant differences were noted, n=40 (Welch’s t Test).

10.7717/peerj.9799/supp-4 Supplemental Information 4 Cilia length determination in uninduced and OS differentiated in MC3T3-E1 preosteoblast cells by dual ciliary labeling. (A) ****Representative images of primary cilium in MC3T3-E1 at day 0 uninduced without serum starvation (day 0 uninduced no ss), day 0 and 7 uninduced and in 7 day OS media stimulated cells. Cilia were co-immunolabeled with markers, acetylated α tubulin (red) and Arl13b (green); nuclei were labeled by DAPI (blue). (B) Ciliary length was measured for each marker and condition and no significant differences were noted, n=40 (Welch’s t Test).

10.7717/peerj.9799/supp-5 Supplemental Information 5 Lower magnification images of primary cilia during chondrogenic differentiation in ATDC5 cells. Primary cilia were labeled with acetylated α tubulin (green), while nuclei were stained with DAPI (blue). Scale bar: 5 μm. Images were obtained at 40X magnification.

10.7717/peerj.9799/supp-6 Supplemental Information 6 Lower magnification views of primary cilia during osteogenic differentiation in C3H10T1/2 and MC3T3-E1 cells. Primary cilia were labeled with acetylated α tubulin (green), while nuclei were stained with DAPI (blue). Images were obtained at 40X magnification. Scale bar: 5 μm.

10.7717/peerj.9799/supp-7 Supplemental Information 7 Cilia length variability in the absence of osteogenic and chondrogenic differentiation. Two sets of undifferentiated cells were considered at day 0, non-serum starved (day 0 uninduced no ss) and starved (day 0 uninduced). All other day matched uninduced cells were starved. (A) Cilia length in day 14 uninduced ATDC5 monolayers was significantly longer than at day 0 uninduced no ss, n=110-152 (**** p<0.0001, One-way ANOVA followed by Tukey’s post hoc analysis). (B) In C3H10T1/2 cells, primary cilia were significantly shorter and longer at 7 and 14 days, respectively compared to day 0 uninduced no ss, n=111-155 (** p<0.01, **** p<0.0001, One-way ANOVA followed by Tukey’s post hoc analysis). (C) Primary cilia length was significantly increased with starvation in day 0 uninduced MC3T3-E1 cells; at days 7, 14 and 21 uninduced cells displayed significantly longer cilia compared to day 0 uninduced, n=101-155 (** p<0.01, **** p<0.0001, One-way ANOVA followed by Tukey’s post hoc analysis).

10.7717/peerj.9799/supp-8 Supplemental Information 8 Primary cilia prevalence was not altered with chondrogenic and osteogenic differentiation in ATDC5, C3H10T1/2 and SAG treatment. All differentiated and undifferentiated cells were serum starved (ss) except single set of uninduced cells at day 0 (day 0 uninduced no ss). No significant variation in primary cilia frequencies were observed in (A) ATDC5 with CH differentiation, n=317-527, and OS induction in (B) C3H10T1/2, n=311-432 and (C) SAG treatment with or without OS differentiation over a 7 day period in C3H10T1/2 cells, n=311-432 (One-way ANOVA followed by Tukey’s post hoc analysis).

10.7717/peerj.9799/supp-9 Supplemental Information 9 Raw data for mineralization levels by Alizarin red staining in osteogenic and chondrogenic differentiation of C3H10T1/2, MC3T3-E1 and ATDC5 cells.

10.7717/peerj.9799/supp-10 Supplemental Information 10 Raw data for transcript levels of *Alp*, *Runx2* and *Ptch1* during osteogenesis in C3H10T1/2.

10.7717/peerj.9799/supp-11 Supplemental Information 11 Raw data for *Alp* and *Runx2* gene expression during osteogenic differentiation in MC3T3-E1.

10.7717/peerj.9799/supp-12 Supplemental Information 12 Raw data for glycosaminoglycan (GAG) deposition in the ECM following 21 days of chondrogenic differentiation in ATDC5 cells.

10.7717/peerj.9799/supp-13 Supplemental Information 13 Raw data for *Sox9* and *Mmp13* transcript levels following chondrogenic differentiation in ATDC5 cells.

10.7717/peerj.9799/supp-14 Supplemental Information 14 Raw data for primary cilia length and frequency variation during chondrogenesis in ATDC5 cells.

10.7717/peerj.9799/supp-15 Supplemental Information 15 Raw data for primary cilia length estimation by dual markers in uninduced and following chondrogenesis in ATDC5 cells.

10.7717/peerj.9799/supp-16 Supplemental Information 16 Raw data for primary cilia length and incidence during osteogenic differentiation and SAG stimulation in C3H10T1/2 cells.

10.7717/peerj.9799/supp-17 Supplemental Information 17 Raw data for primary cilia length evaluation in uninduced and osteogenic differentiation in C3H10T1/2 by dual marker labeling.

10.7717/peerj.9799/supp-18 Supplemental Information 18 Raw data for primary cilia length and frequency variation during osteogenic differentiation in MC3T3-E1.

10.7717/peerj.9799/supp-19 Supplemental Information 19 Raw data for cilia length determination in uninduced and osteogenic differentiation in MC3T3-E1 by dual marker labeling.

10.7717/peerj.9799/supp-20 Supplemental Information 20 Raw data for *Alp* expression levels following SAG treatment in C3H10T1/2 cells.

10.7717/peerj.9799/supp-21 Supplemental Information 21 Raw data for comparison of viable cell numbers during osteogenic differentiation in C3H10T1/2.

## Additional Information and Declarations

### Data Availability

The following information was supplied regarding data availability: Raw data for all figures are available as Supplemental Files and additional data are available as Supplemental Figures.
