# Supplementary figures and images for: Characterization of primary cilia features reveal cell-type specific variability in in vitro models of osteogenic and chondrogenic differentiation
Source: PeerJ. 2020 Aug 21;8:e9799. doi: 10.7717/peerj.9799 (PMC7444507; doi:10.7717/peerj.9799)

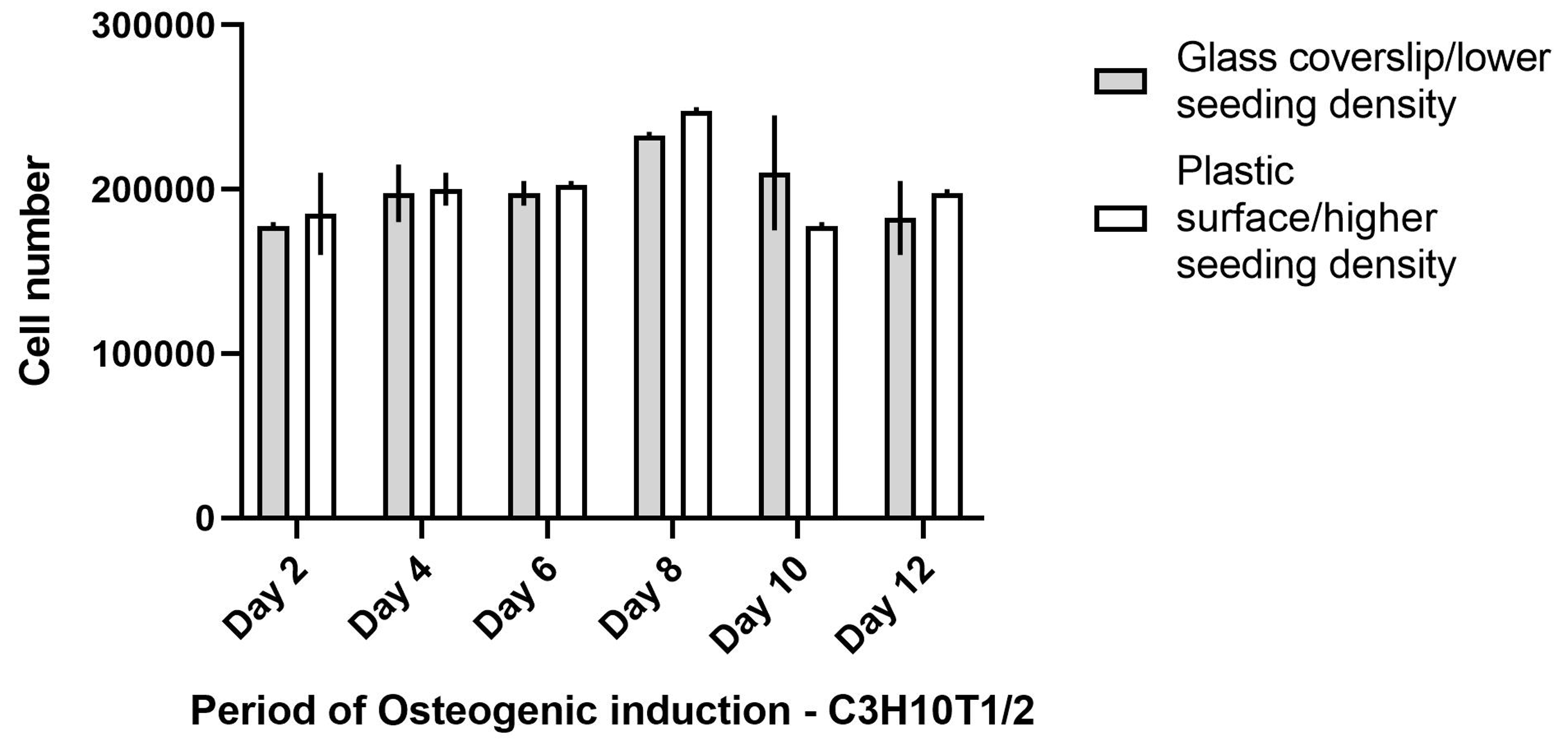

Supplement: Supplemental Information 1 — Cells were seeded at a density of 18000 cm2 and 21000 cm2 on glass and plastic, respectively followed by OS induction. Count of viable cells were estimated by Trypan blue at 2, 4, 6, 8, 10 and 12 days and no significant differences were observed (Two way ANOVA followed by Tukey’s post hoc analysis). [file peerj-08-9799-s001.png]

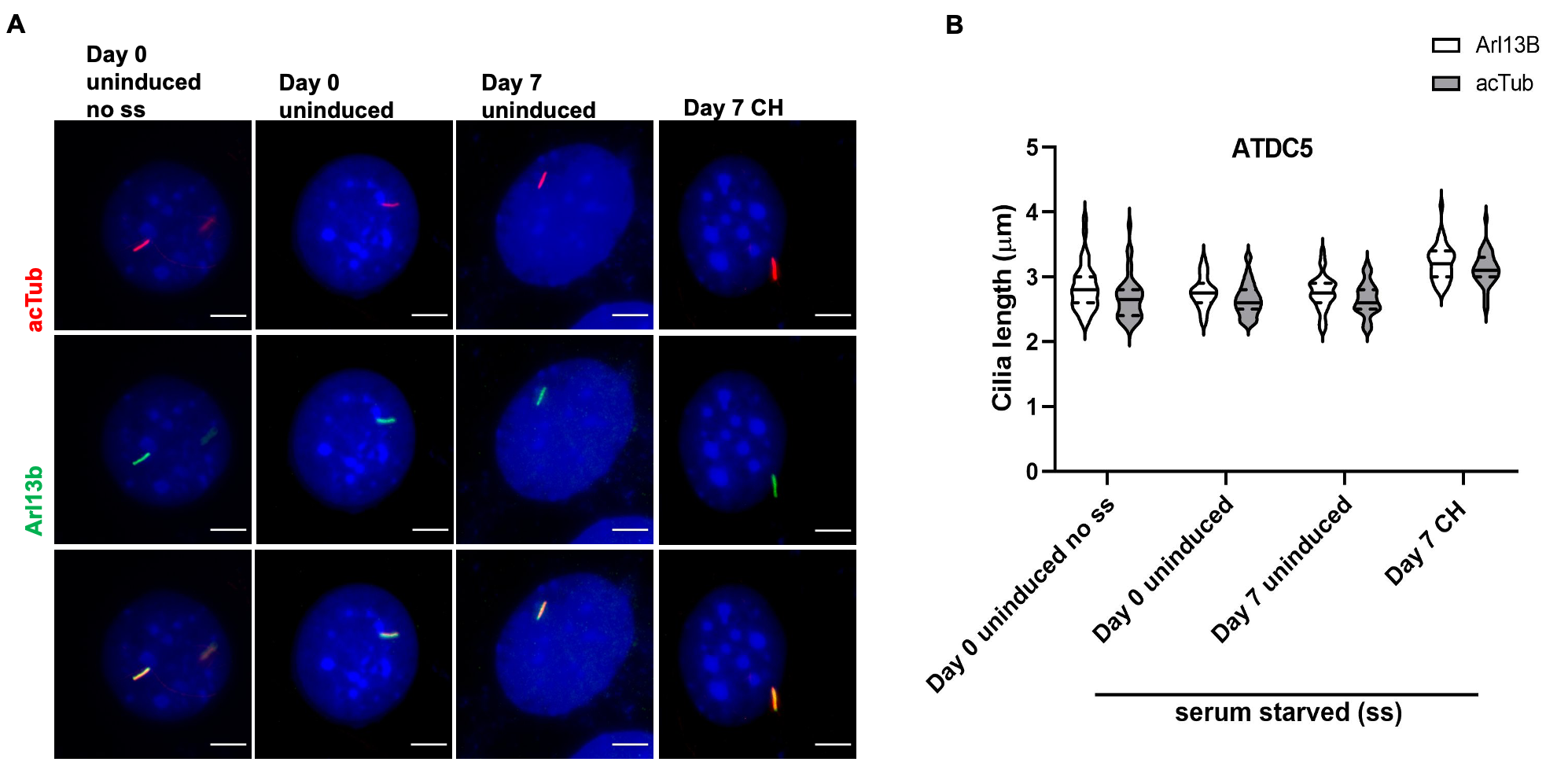

Supplement: Supplemental Information 2 — (A) Representative images of primary cilium in ATDC5 at day 0 uninduced without serum starvation (day 0 uninduced no ss), day 0 and 7 uninduced and in 7 day CH differentiated cells. Cilia were co-immunolabeled with dual markers, acetylated α tubulin (red) and Arl13b (green); nuclei were labeled by DAPI (blue). (B) Ciliary length was measured for each marker and condition and no significant differences were observed, n=40 (Welch’s t Test). [file peerj-08-9799-s002.png]

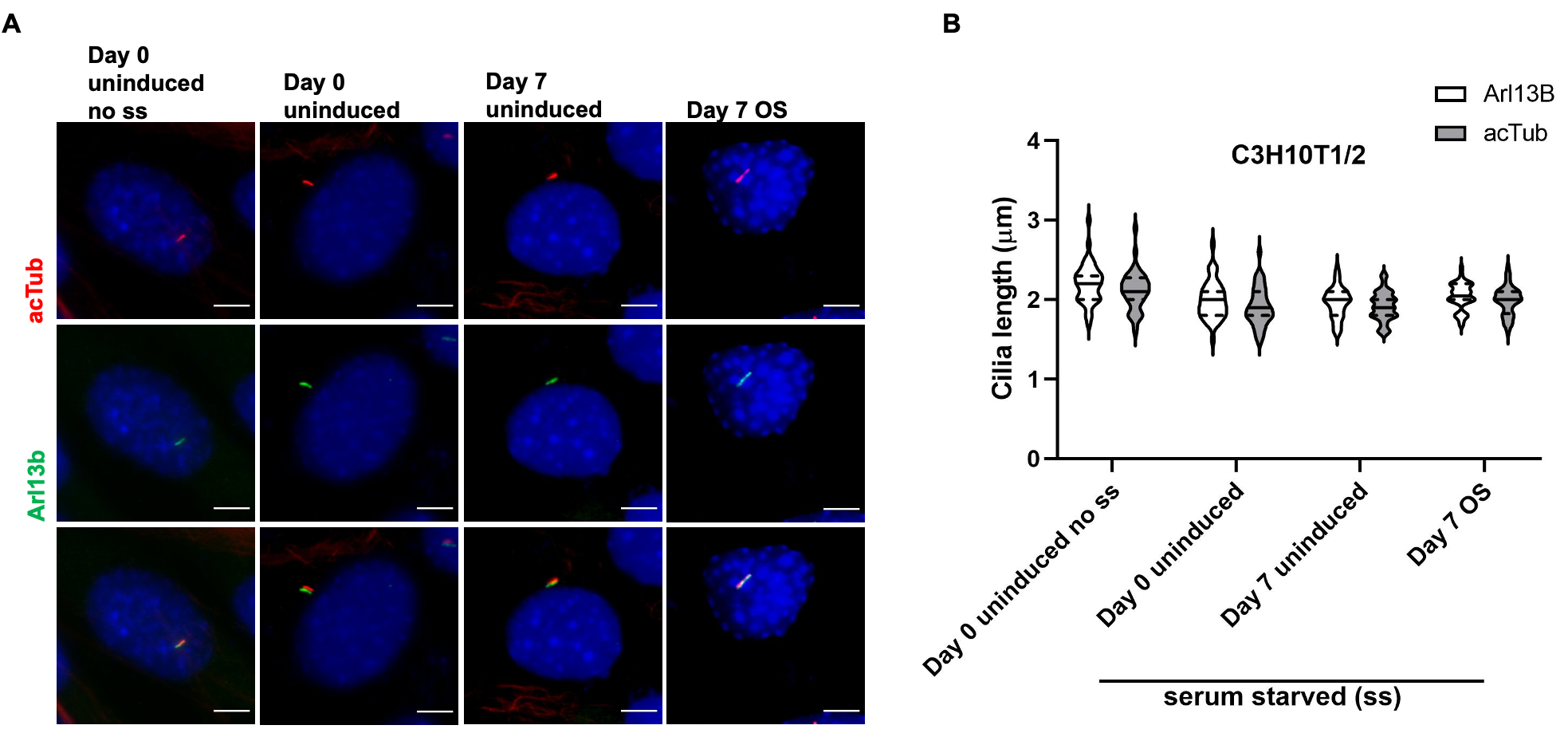

Supplement: Supplemental Information 3 — (A) Representative images of primary cilium in C3H10T1/2 at day 0 uninduced without serum starvation (day 0 uninduced no ss), day 0 and 7 uninduced and in 7 day OS induced cells. Cilia were co-immunolabeled with markers, acetylated α tubulin (red) and Arl13b (green); nuclei were labeled by DAPI (blue). (B) Ciliary length was measured for each marker and condition and no significant differences were noted, n=40 (Welch’s t Test). [file peerj-08-9799-s003.png]

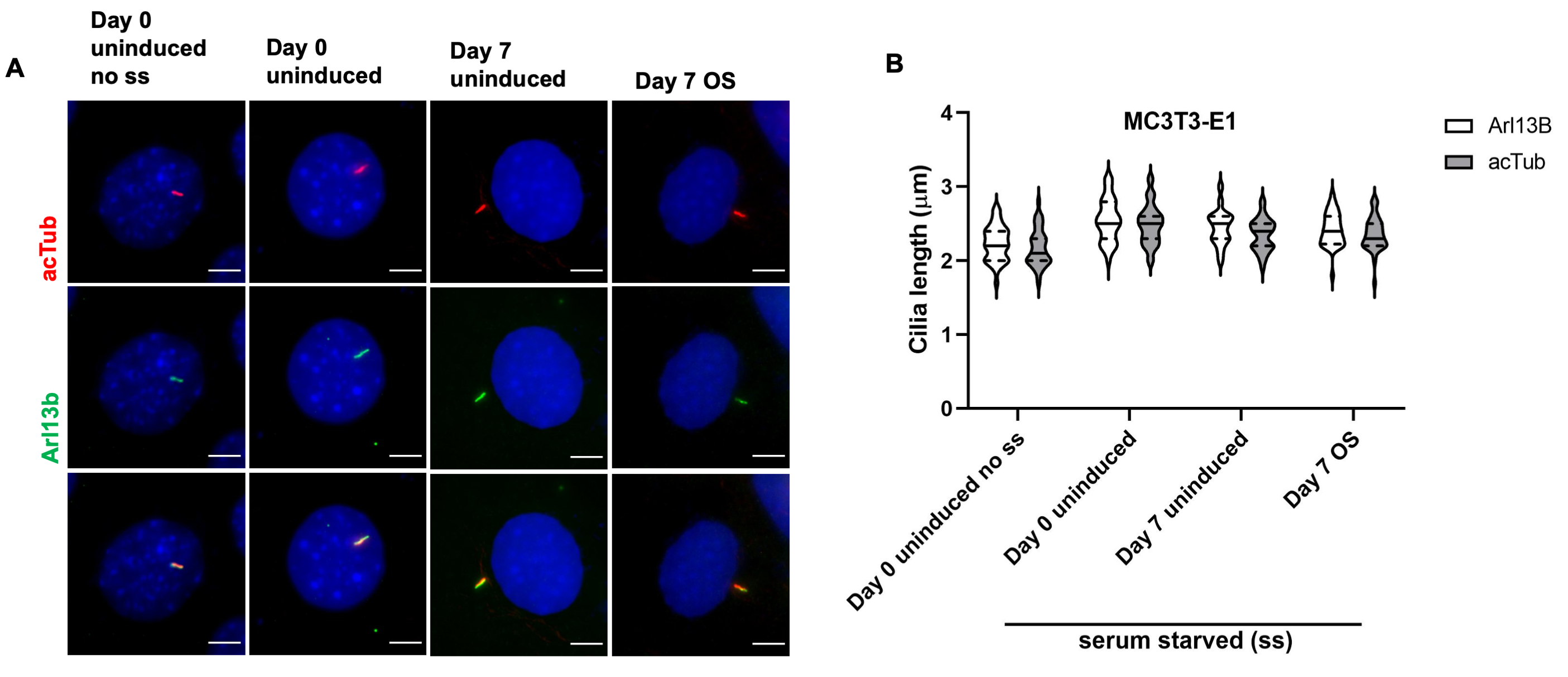

Supplement: Supplemental Information 4 — (A) Representative images of primary cilium in MC3T3-E1 at day 0 uninduced without serum starvation (day 0 uninduced no ss), day 0 and 7 uninduced and in 7 day OS media stimulated cells. Cilia were co-immunolabeled with markers, acetylated α tubulin (red) and Arl13b (green); nuclei were labeled by DAPI (blue). (B) Ciliary length was measured for each marker and condition and no significant differences were noted, n=40 (Welch’s t Test). [file peerj-08-9799-s004.png]

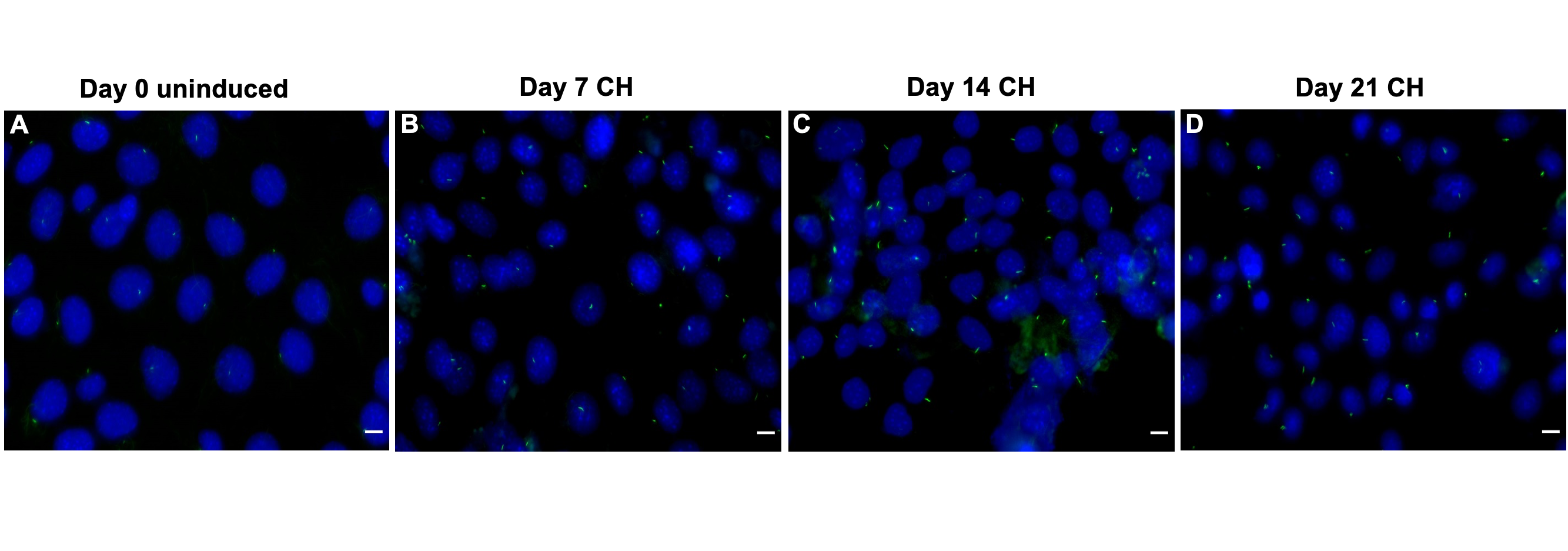

Supplement: Supplemental Information 5 — Primary cilia were labeled with acetylated α tubulin (green), while nuclei were stained with DAPI (blue). Scale bar: 5 μm. Images were obtained at 40X magnification. [file peerj-08-9799-s005.png]

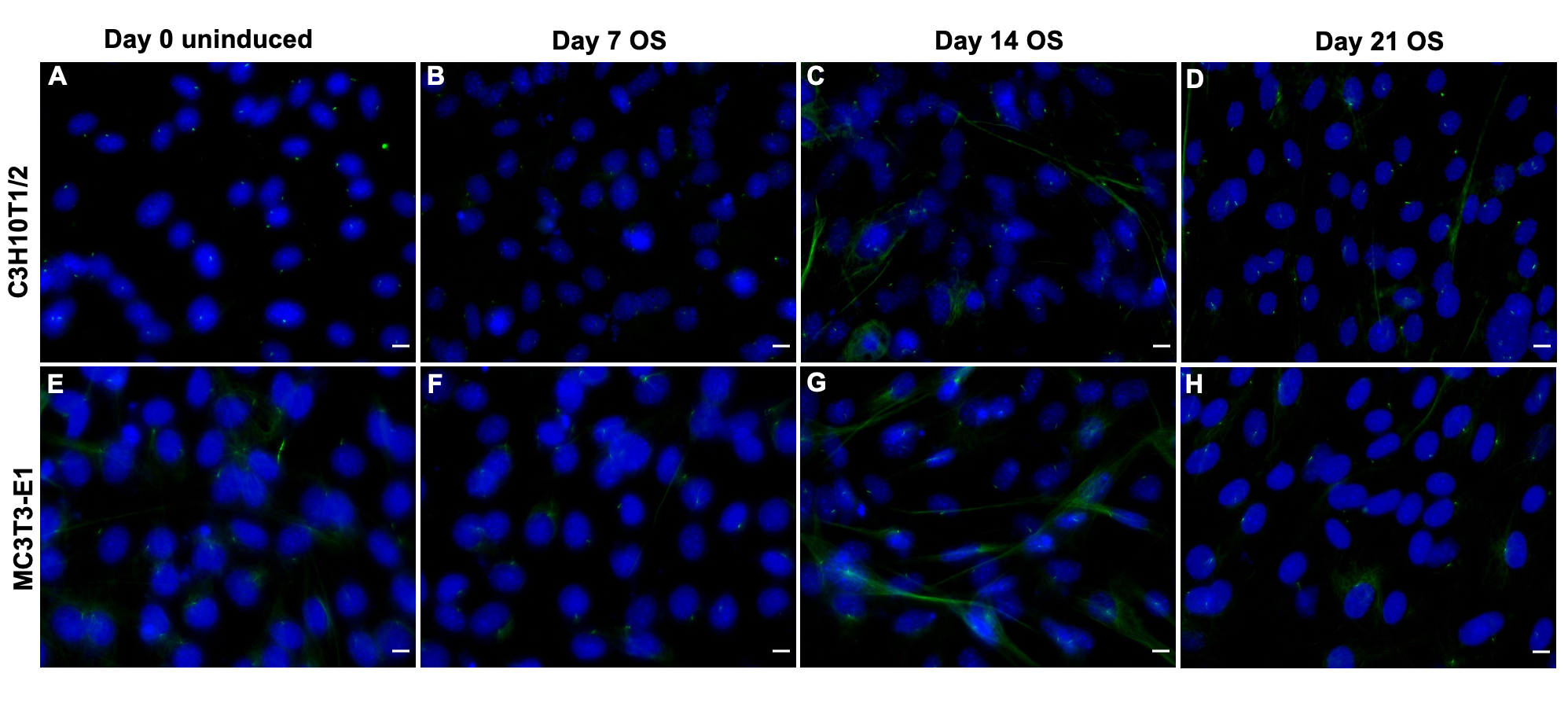

Supplement: Supplemental Information 6 — Primary cilia were labeled with acetylated α tubulin (green), while nuclei were stained with DAPI (blue). Images were obtained at 40X magnification. Scale bar: 5 μm. [file peerj-08-9799-s006.png]

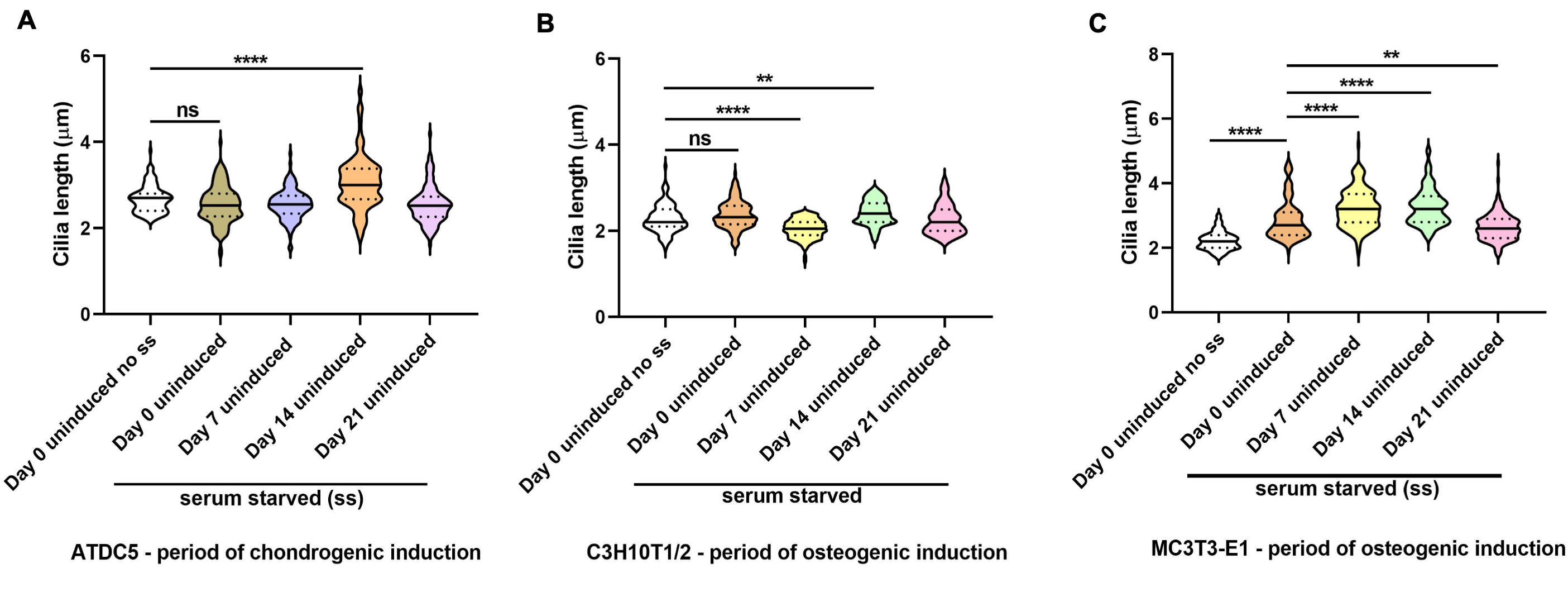

Supplement: Supplemental Information 7 — Two sets of undifferentiated cells were considered at day 0, non-serum starved (day 0 uninduced no ss) and starved (day 0 uninduced). All other day matched uninduced cells were starved. (A) Cilia length in day 14 uninduced ATDC5 monolayers was significantly longer than at day 0 uninduced no ss, n=110-152 (**** p<0.0001, One-way ANOVA followed by Tukey’s post hoc analysis). (B) In C3H10T1/2 cells, primary cilia were significantly shorter and longer at 7 and 14 days, respectively compared to day 0 uninduced no ss, n=111-155 (** p<0.01, **** p<0.0001, One-way ANOVA followed by Tukey’s post hoc analysis). (C) Primary cilia length was significantly increased with starvation in day 0 uninduced MC3T3-E1 cells; at days 7, 14 and 21 uninduced cells displayed significantly longer cilia compared to day 0 uninduced, n=101-155 (** p<0.01, **** p<0.0001, One-way ANOVA followed by Tukey’s post hoc analysis). [file peerj-08-9799-s007.png]

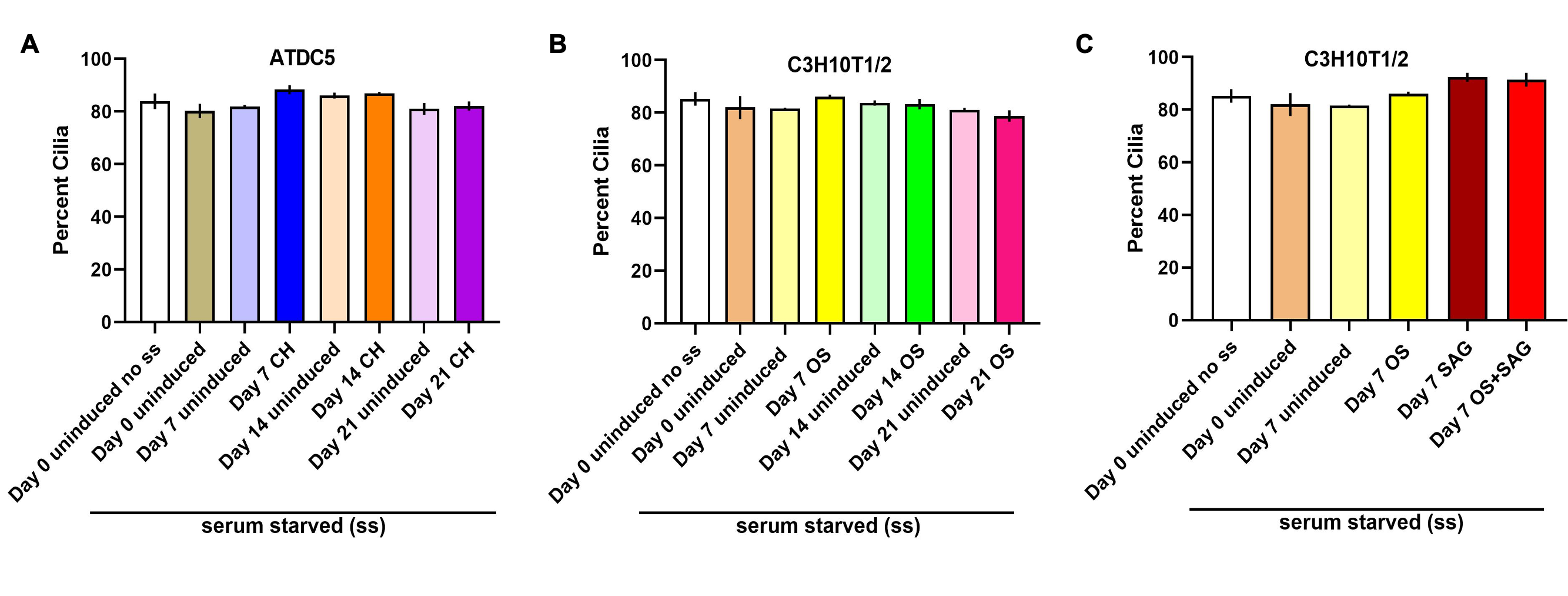

Supplement: Supplemental Information 8 — All differentiated and undifferentiated cells were serum starved (ss) except single set of uninduced cells at day 0 (day 0 uninduced no ss). No significant variation in primary cilia frequencies were observed in (A) ATDC5 with CH differentiation, n=317-527, and OS induction in (B) C3H10T1/2, n=311-432 and (C) SAG treatment with or without OS differentiation over a 7 day period in C3H10T1/2 cells, n=311-432 (One-way ANOVA followed by Tukey’s post hoc analysis). [file peerj-08-9799-s008.png]
